# Defining core competencies for advanced therapy medicinal products translation

**DOI:** 10.3389/fdgth.2026.1707741

**Published:** 2026-02-12

**Authors:** Elena Guillen, Ana Hidalgo-Simon

**Affiliations:** 1 Leiden University Medical Center, Leiden, Netherlands; 2 The Novo Nordisk Foundation Center for Stem Cell Medicine (reNEW), Leiden, Netherlands

**Keywords:** advanced therapy medicinal products (ATMPs), competencies, gene and cell therapies, regulatory science, training, translation, workforce

## Abstract

**Background:**

Translating Advanced Therapy Medicinal Products (ATMPs) from research to clinical application requires a specialized set of priority topic areas that is not yet systematically defined.

**Methods:**

We employed a multi-phase approach combining a targeted literature review with a structured expert consensus survey. Selected experts evaluated a set of 35 preselected competencies across seven thematic areas using an anonymous online questionnaire, categorizing them as Fundamental, Ancillary, or Less Important for ATMPs translation.

**Results:**

Concordance was high, with 16 competencies reaching the ≥75% consensus threshold as Fundamental. These covered regulatory strategy, manufacturing scalability, quality management, clinical translation, and cross-disciplinary collaboration.

**Conclusions:**

This study establishes a validated set of ATMP-specific competencies that can serve as the basis for targeted training programs. Embedding these into education and professional development will help build a workforce capable of navigating the complex and constantly evolving translational needs of ATMPs.

## 1 Introduction

Advanced therapy Medicinal Products (ATMPs) are medicinal products based on genes, tissues, or cells. They are often classified as ‘cell and gene therapy (CGT)’ products, although their definition remains fluid across countries and regulatory frameworks (1, 2). ATMPs have revolutionized healthcare due to their potential to treat unmet medical needs and orphan diseases. However, despite significant investment and progress in ATMPs research, only a small portion achieve marketing authorization and sustained market presence,. This gap reflects not only technical and regulatory challenges, but also a shortage of skills among healthcare and research professionals required to translate ATMPs effectively into patient care.

Success in translating ATMPs depends on the seamless integration of multiple scientific, clinical, regulatory and operational components. This complexity necessitates a “team science” approach, bringing together multidisciplinary expertise to support the progression of ATMPs across development stages, from early research through clinical application (3–5). Intrinsically patient-focused and driven by clinical application, the ultimate goal of translation science is to advance public health outcomes (6).

The current translational framework is better understood as a map rather than a linear pipeline. It is a multi-step and recursive process that includes a spectrum of research, implementation, manufacturing and post-market surveillance (3, 4). Despite this conceptual evolution, multiple authors have noted that translational capacity has lagged behind advances in basic research over the past four decades, resulting in inefficiencies in moving innovations into clinical practice (6).

In this paper we identify the key specialist competencies necessary for the effective translation and implementation of ATMPs. Moreover, via targeted literature review and expert consensus-building through a structured expert consensus survey, we outline a core set of competences necessary to advance ATMPs from research to clinical practice.

## 2 Methods

While ATMPs are diverse, domains of expertise essential for successful translation can be identified.

For this, we employed a multi-phase approach incorporating targeted review and expert consensus-building through a structured expert consensus survey.

Our first step involved a targeted review of translational science literature to collate a preliminary longlist of competencies and organize them thematically. We specifically searched for competencies relevant to both general translation and those uniquely required for ATMP development. Sources included peer-reviewed publications, regulatory guidelines, white papers, and reports from relevant ATMP organizations such as ARM (Alliance for Regenerative Medicine) and EATRIS (European Infrastructure for Translational Medicine) (7, 8). From this, we compiled preliminary selection of competencies, based on their alignment with ATMP-specific challenges, including regulatory complexity, manufacturing scalability, or intellectual property issues. Identified competencies were categorized into thematic areas. Other less specific competencies, are considered as a preamble to translational science as a whole and therefore have not been considered.

The seven thematic areas chosen were Drug Discovery and Development, Preclinical Development (3 competencies each), Clinical Development and Trial Innovation (2 competencies), Technology and Analytical Tools (5 competencies), Manufacturing/Chemistry, Manufacturing and Controls (CMC) (8 competencies), Regulatory Process and Compliance, and Economic Considerations (7 competencies each). A complete list of all 35 competencies, together with their definitions, is provided in the Supplementary Information. To reduce the risk of omitting relevant skills, participants were also given open-text fields to propose additional competencies beyond the pre-selected list.

Secondly, a structured expert consensus survey was conducted to validate and further refine the preselected competencies. The survey was distributed anonymously via REDCap to a purposively selected panel of experts with recognized experience in ATMP development, manufacturing, regulation, and translation. Participants were identified through leading advanced therapy organizations, established professional networks, ensuring representation from academia, industry, and regulatory bodies. Each participant was asked to evaluate every competency based on its relevance and importance for ATMP translation. The questionnaire categorized competencies into three levels of importance: Fundamental (i.e., essential competencies required for successful ATMP translation), Ancillary (i.e., supportive competencies that enhance translational effectiveness but are not strictly essential) and Less Important (i.e., competencies that, while valuable, were deemed lower priority in the ATMP translation priorities). Open answer fields allowed participants to provide additional comments. Not all participants rated every competency, resulting in occasional missing data. Percentages were calculated based on the number of participants who provided a rating for each item. Moreover, although the focus of the structured expert consensus survey is on technical skills, an open answer field on soft skills were also included. Brief demographic information regarding area of work was also collected in order to ensure a balanced representation of participants. Efforts were made to ensure a higher range of academic and industry participants compared to regulators or service providers, as these areas are directly involved in the development of ATMPs, where there is a notable shortage of individuals specifically trained in ATMP translation.

As the main focus of this structured expert consensus survey is to define the set of competencies considered most relevant for ATMP translation, experts were asked to rate each pre-identified competency into fundamental, ancillary and less important. A threshold of ≥75% of participants considering a competency fundamental was selected for inclusion in the final priority topic areas. This cutoff reflects a conservative level of agreement commonly used in expert consensus studies. We selected 75% as it represents a balance between inclusivity and stringency: lower thresholds (e.g., 70%) could include competencies with more variable support, while higher thresholds (e.g., 80%) risk excluding areas broadly agreed upon but with some heterogeneity. Competencies not meeting this threshold were not considered irrelevant but classified as ancillary or context-dependent, reflecting greater variability in expert opinion. Moreover, to assess the robustness of this choice, a sensitivity analysis was conducted. Applying an 80% threshold would have excluded two competencies that were otherwise strongly supported (Reimbursement Assessment & Market Access; Biobanking & Cell/Tissue Sourcing), while a 70% threshold would have included three additional competencies (Funding Strategy & Sustainability; Regulatory Product Classification; Regulatory Science & Systems Overview). These additional competencies largely reflect contextual or system-level considerations, supporting their classification as ancillary rather than universally fundamental. In addition, open-text fields allowed participants to suggest further competencies not initially listed.

In this manuscript, we define competencies as the applied knowledge and skills required to perform key functions in ATMP development.

## 3 Results

A total of 25 experts participated in the structured expert consensus survey. Table 1 shows an overview of the participants setting of work.

**Table 1:** Setting of work of participants of the structured expert consensus survey.

Participant setting of work	Number of participants (%)
Academia/Hospital	9 (36%)
Industry	6 (24%)
Patient Organization	4 (16%)
Other	3 (12%)
	1 CDMO/Biotech
	1 Non-profit blood bank
	1 Regulatory Agency
Not specified	3 (12%)

Participants were asked about 35 competencies, divided into 7 thematic areas. The complete results of the structured expert consensus survey can be found in Table 2. Of the proposed list, 16 (45%) competencies were ranked as Fundamental by more than 75% of participants.

**Table 2:** Percentage of respondents rating each competency as Fundamental, Ancillary or Less Important. Percentages are calculated based on the number of participants who rated each competency. Where applicable, missing responses are reflected in the denominators.

Thematic Area	Competency	% of respondents rating each competency as Fundamental, Ancillary or Less Important
**DRUG DISCOVERY AND DEVELOPMENT**	*In Vitro* Models for Toxicology and Mechanism Studies	F 84% (21/25)
		A 16% (4/25)
		L 0% (0/25)
	Biomarkers and Surrogate Endpoints	F 56·6% (13/23)
		A 39·1% (9/23)
		L 4·3% (1/23)
	Clinical Proof of Concept & Mechanism	F 84% (21/25)
		A 12% (3/25)
		L 4% (1/25)
**PRECLINICAL**	Linking Preclinical Results to Clinical Viability	F 92% (23/25)
		A 8% (2/25)
		L 0% (0/25)
	Preclinical Models and Alternatives	F 64% (16/25)
		A 32% (8/25)
		L 4% (1/25)
	Good Laboratory Practices (GLP) Research Principles and Applications	F 84% (21/25)
		A 16% (4/25)
		L 0% (0/25)
**CLINICAL DEVELOPMENT & TRIAL INNOVATION**	Innovative Clinical Trial Designs	F 60% (15/25)
		A 32% (8/25)
		L 8% (2/25)
	Long-Term Follow-Up in Trials	F 68% (17/25)
		A 28% (7/25)
		L 4% (1/25)
**TECHNOLOGY & ANALYTICAL TOOLS**	Statistical Approaches for Novel Trial Designs	F 52% (13/25)
		A 40% (10/25)
		L 8% (2/25)
	Modeling & In Silico Methods	F 48% (12/25)
		A 40% (10/25)
		L 12% (3/25)
	Biobanking & Cell/Tissue Sourcing	F 76% (19/25)
		A 20% (5/25)
		L 4% (1/25)
	Emerging Technologies in Regulatory Science	F 28% (7/25)
		A 68% (17/25)
		L 4% (1/25)
	Platform Technology Integration	F 52% (13/25)
		A 44% (11/25)
		L 4% (1/25)
**MANUFACTURING/CHEMISTRY, MANUFACTURING AND CONTROLS (CMC)**	Good Manufacturing Practice (GMP) Compliance	F 96% (24/25)
		A 4% (1/25)
		L 0% (0/25)
	Analytical Methods & Quality Control testing	F 96% (24/25)
		A 4% (1/25)
		L 0% (0/25)
	Critical Quality Attributes)	F 91·7% (22/24)
		A 8·3% (2/24)
		L 0% (0/24)
	Risk Management and Contingency Planning	F 84% (21/25)
		A 16% (4/25)
		L 0% (0/25)
	CMC Quality Assurance Implementation	F 92% (23/25)
		A 8% (2/25)
		L 0% (0/25)
	Process Optimization & Tech Transfer	F 56% (14/25)
		A 40% (10/25)
		L 4% (1/25)
	Automation Evaluation & Implementation	F 44% (11/25)
		A 52% (13/25)
		L 4% (1/25)
	Process Scalability and Batch Consistency	F 88% (22/25)
		A 12% (3/25)
		L 0% (0/25)
**REGULATORY PROCESS & COMPLIANCE**	Regulatory Strategy Development	F 88% (22/25)
		A 12% (3/25)
		L 0% (0/25)
	Regulatory Science & Systems Overview	F 72·7% (18/25)
		A 28% (7/25)
		L 0% (0/25)
	Regulatory Product Classification	F 72% (16/25)
		A 28% (7/25)
		L 8% (2/25)
	Law & Policy Evaluation	F 44% (10/23)
		A 48% (11/23)
		L 8% (2/23)
	Post-Market Compliance & Risk Mitigation	F 68% (17/25)
		A 24% (6/25)
		L 8% (2/25)
	Risk-Benefit Assessment Innovations	F 84% (21/25)
		A 16% (4/25)
		L 0% (0/25)
	Ethical Considerations in Research & Use	F 84% (21/25)
		A 12% (3/25)
		L 4% (1/25)
**ECONOMIC CONSIDERATIONS**	Intellectual Property Strategy	F 64% (16/25)
		A 36% (9/25)
		L 0% (0/25)
	Economic Viability of Novel Medical Products	F 80% (20/25)
		A 12% (3/25)
		L 8% (2/25)
	Funding Strategy & Sustainability	F 72% (18/25)
		A 28% (7/25)
		L 0% (0/25)
	Global Landscape & Commercialization Pathways	F 60% (15/25)
		A 40% (10/25)
		L 0% (0/25)
	Reimbursement Assessment & Market Access	F 79% (19/24)
		A 20·8% (5/24)
		L 0% (0/24)

Highest concordance was observed for competencies in the thematic area of manufacturing/CMC, where six out of the eight proposed competencies were ranked as Fundamental by more than 84% of participants. The highest concordance was achieved for Good Manufacturing Practice (GMP) Compliance and Analytical Methods & Quality Control testing, with 24 (96%) participants agreeing. This was closely followed by CMC Quality Assurance Implementation (23 participants, 92%), Linking Preclinical Results to Clinical Viability (23 participants, 92%), Critical Quality Attributes (22 participants, 91·7%), Process Scalability and Batch Consistency (22 participants, 88%) and Regulatory Strategy Development (22 participants, 88%).

Drug Discovery and Development and Preclinical thematic areas also showed strong consensus for specific competencies, with *in vitro* Models for Toxicology and Mechanism Studies, Clinical Proof of Concept & Mechanism, and Good Laboratory Practices (GLP) each rated Fundamental by 21 participants (84%).

The lowest concordance for Fundamental ranking was seen in Emerging Technologies in Regulatory Science, with 7 participants (28%) rating it as Fundamental; it was instead most frequently considered Ancillary (17 participants, 68%). Other competencies with less than half of participants rating them as Fundamental included Modeling & In Silico Methods (48%, 12 participants), Law & Policy Evaluation (44%, 10 participants), and Automation Evaluation & Implementation (44%, 11 participants).

The whole thematic area of Clinical Development & Trial Innovation did not achieve the threshold of 75%.

Across the dataset, none of the competencies were ranked as Less Important by more than 3 participants. In fact, 21 out of the 35 proposed competencies were not ranked as Less Important by any participant.

The open-text fields, designed to capture competencies not included in the pre-selected set, did not reveal any substantial gaps. A few additional suggestions were provided, but each was mentioned only once and served to complement rather than replace the existing set. These included: basic science and innovation in scientific developments, centralized and decentralized manufacturing, advanced immunogenicity and safety profiling for gene therapy, good distribution practices and supply chain management, awareness of GMP legislation and its interplay with substance of human origin legislation, and registries and patient-reported outcome methods.

The soft skill that was most repeated by participants as necessary was communication, suggested by a total of 11 participants, followed by critical thinking and collaboration/teamwork (repeated by 6 participants, respectively) and adaptability/learning agility, strategic thinking and problem-solving (repeated by 4 participants). The entire list of suggested soft skills is outlined in Table 3.

**Table 3:** Recomplicated and grouped answers of participants regarding soft skills.

SOFT SKILLS (grouped)		Answers of participants
Communication & Collaboration	Communication	Influential/Tailored communication (audience dependent) Interdisciplinary Communication Cross-functional Communication Communication is always key Communication (as cross-functional, multidisciplinary teamwork is needed) Stakeholder engagement
	Collaboration & Teamwork	Cross-functional collaboration Collaborative teamwork Teamwork/collaborative spirit Collaboration An ability to admit one does not know everything and can ask for assistance Multidisciplinarity, teamwork Teamwork
	Patient-centric Collaboration	Patient involvement Patient involvement in ATMP life-cycle (esp. economic evaluations)
Thinking Skills & Insight	Critical Thinking	Critical thinking (repeated frequently) Critical thinking & adaptability Attention to detail Ethical judgment
	Creative/Strategic Thinking	Strategic thinking/Strategic leadership Strategic thinking with scientific insight Think outside the box Innovative thinking/Creative thinking Understanding of business scaling
	Problem Solving	Problem-solving mindset Problem-solving across regulatory and scientific barriers Risk prevention Freedom to operate challenge
	Scientific & Systems Insight	Scientific literacy Focus on the final goal: to treat the patient
Adaptability & Growth	Flexibility, self-awareness, and continuous improvement.	Adaptability/Learning agility Open-mindedness Long-term resilience Commitment to a common mission Ability to ask for help/humility Results focus Planning and organizing (project management) Resilience Leadership The funding cliff for translation

## 4 Discussion

### 4.1 Alignment of findings with existing evidence

This structured expert consensus survey engaged selected experts to prioritize 35 pre-identified competencies grouped into seven thematic areas focused on the translation of ATMPs. Our results revealed no major gaps, with only a few complementary suggestions, confirming the adequacy of our initial pre-selection. Concordance was generally high, with nearly half of the competencies (16 out of 35) rated as Fundamental for successful ATMP translation. This and the broad absence of “Less Important” ratings suggest a shared mental model among stakeholders regarding the core technical backbone required for ATMP translation. This aligns with recent CGT workforce and training mappings that highlight convergent priorities across academia, hospitals, and industry (e.g., GMP, Quality Assurance/Quality Control, process and scale-up) (9). The strongest agreement was concentrated in Manufacturing/CMC, with six of the eight competencies in this thematic area rated Fundamental by more than 84% of participants. This dominance of CMC reflects well-known translation bottlenecks in ATMPs: GMP readiness, robust analytical methods, defined critical quality attributes, and scalable, consistent processes, and is in line with multiple sector analyses and surveys (10, 11). Various authors have similarly identified manufacturing, Quality Control, and analytical development as the most acute capability needs and the most limiting steps for translation and scale-up. This is particularly challenging for academic developers, where lack of understanding of quality terminology and regulatory expertise in general is scarce (12, 13).

Alongside CMC, competencies such as regulatory strategy, risk–benefit assessment, and ethical considerations also reached strong support. This reflects growing calls for early and integrated regulatory science in translational programs, where proactive engagement with regulators can streamline pathways, reduce uncertainty, and align with evolving guidance. Addressing ethical considerations early further ensures that patient safety and societal acceptability are embedded into translational decision-making (14, 15).

Consensus was also observed around competencies that strengthen preclinical-clinical bridging, such as the use of *in vitro* models, clinical proof of concept, and adherence to GLP, addressing reproducibility and comparability concerns raised in ATMP development (16).

In contrast, the Clinical Development & Trial Innovation thematic area did not reach the threshold for any competency to be classified as Fundamental. This likely reflects the perception that expertise in innovative trial design or long-term follow-up, is more domain-specific or context-dependent rather than universally core to ATMP translation. Unlike manufacturing or regulatory competencies, which are critical for all ATMP programs, clinical trial expertise may vary depending on the therapy modality, trial setting, or institutional support. As such, participants may have rated these competencies as Ancillary, recognizing their importance in specific scenarios without considering them essential across all translational programs. This may suggest that certain competencies are situationally critical, reinforcing the need for multidisciplinary teams where specialized clinical trial knowledge complements broader translational expertise. In addition, clinical competencies are likely already established in groups that have reached an advanced stage of ATMP development, making them less relevant as universally fundamental in this consensus.

Competencies related to intellectual property strategy and economic viability/funding showed comparatively lower consensus. This pattern is also consistent with the literature: financing translational “valleys of death,” freedom-to-operate, and market access strategy vary widely by jurisdiction, asset class, and business model (17). As a result, teams may view these skills as situational or specialist rather than universally core. Nonetheless, high consensus in economic competencies such as viability assessment and market access planning, acknowledge that translation is only complete when the therapy reaches patients sustainably, emphasizing health economics and reimbursement planning as critical translational enablers (18).

Competencies such as emerging technologies in regulatory science, automation evaluation, and modeling & in silico methods received lower consensus, likely because they are not perceived as core or universally applicable to all ATMP modalities. While these innovative and specialized approaches hold promise for improving cost-effectiveness, process optimization, and scalability, their integration into ATMP development remains context-dependent and often product-specific. Similar observations have been reported in competency frameworks for other complex biotherapeutics, where foundational regulatory, manufacturing, and quality-related competencies consistently outrank emerging or highly specialized technical skills in perceived importance (18). This suggests that, although desirable, these advanced capabilities are best viewed as enablers rather than prerequisites for successful ATMP translation. Lower agreement on platform technology integration and emerging technologies likely reflects uneven exposure and still-evolving regulatory guidance. While platforms (e.g., vector backbones, modular unit ops) promise comparability and speed, practical know-how and standards remain heterogeneous across organizations and regulators (19). Recent reviews and guidance efforts point to increasing structure (e.g., hospital ATMP Quality Management Systems and education frameworks), but practice is still catching up (20). Although an overall definition of each competency was provided in the structured expert consensus survey, the lack of consensus in these competencies may also reflect potential lack of clarity in the terminology used (e.g., emerging technologies in regulatory science are inherently broad).

Regarding soft skills, respondents most frequently highlighted communication (11 mentions) and then critical thinking and collaboration/teamwork (6 each), followed by adaptability/learning agility, strategic thinking, and problem-solving (4 each). This mirrors team-science literature in translational research, such as the “Fundamental Characteristics of a Translational Scientist” described by Gilliland et al., including the Boundary Crosser, Skilled Communicator, Team Player, and Process Innovator (15). Participants emphasized cross-functional communication, multidisciplinary teamwork, resilience, creativity, and humility—traits essential for navigating ATMP translation's complex, high-stakes environment. This alignment reinforces that successful translation demands not only deep technical expertise but also the mindset and interpersonal abilities to work across disciplines, innovate within constraints, and maintain patient-focused goals. Of note, the category with the highest number of entries was adaptability and growth; flexibility, in its many aspects, was the most frequently represented soft skill in our sample.

### 4.2 Drivers of the need for specialized competencies for the translation of ATMPs

Building from the existing translational science and regulatory science competencies, we believe the successful translation of ATMPs requires a more specialized and integrated set of competencies than what is currently available. This need is driven by several irrelated factors. First, the unique challenges and complexity of ATMPs create substantial demands that current training modules are not fully equipped to address. This gap slows the progress of the entire ecosystem and limits their potential to deliver meaningful clinical outcomes. For instance, cell therapies (often referred to as “living drugs”) require specialized manufacturing, handling, and administration. Because they are frequently customized for individual patients and delivered in tightly controlled settings, their scalability is further complicated. Second, the rapidly evolving technical and basic science environment places additional demands on professionals. The rapid pace of ATMP development requires advances in the other disciplines that support their translation to the clinic. One critical area is regulatory science, where the creation of specific regulatory guidelines and definitions is essential to established harmonized regulatory oversight and increased certainty. However, drafting of regulatory guidance takes time and shared efforts, a process that in many cases is slower than the emergence of new technologies. Third, there are notable shortages in certain knowledge areas critical to ATMP translation. Most traditional biomedical programs focus on basic research or clinical practice without fully addressing the unique requirements of translational science, as noted by other authors (3). Additionally, emerging fields like gene therapy and cell therapy are often underrepresented in training curricula, which limits the workforce's preparedness to bring these therapies to market efficiently. Examples include bioinformatics, intellectual property, market analysis, GMP compliance, manufacturing processes or scalable production methods. Current educational and training frameworks, both graduate and post-graduate realms, often fail to prepare professionals adequately, as they offer very little or no depth in such specialized areas. Moreover, most professionals are often forced to rely on disparate and fragmented sources to acquire this knowledge, which further complicates the learning process. Finally, effective translation requires a highly multidisciplinary approach**.** Beyond scientific expertise, effective patient access requires early understanding of regulatory requirements, as well as strategies for overcoming financial and intellectual property obstacles. In many cases, it is the lack of coordination across these areas—not scientific limitations—that proves to be the primary blocking point preventing promising therapies from reaching patients. The absence of integration reinforces the misleading notion of a linear development path, where research, regulation, financing, and patient access are treated as consecutive rather than interconnected processes. Professionals must develop cross-disciplinary knowledge and be fluent in the “languages” of various domains—such as manufacturing, clinical research, health technology assessment, market access, and legal frameworks—to ensure effective communication and understanding among all stakeholders. Moreover, a skilled professional in ATMPs translation should be able to move between different domains, e.g., academia, industry, regulation. Therefore, this requires training that bridges traditional disciplinary boundaries, equipping professionals with the tools needed to guide therapies through every stage, from research to patient care as a continuity.

### 4.3 Identified key set of specialized competencies for ATMP translation

**Figure 1:**
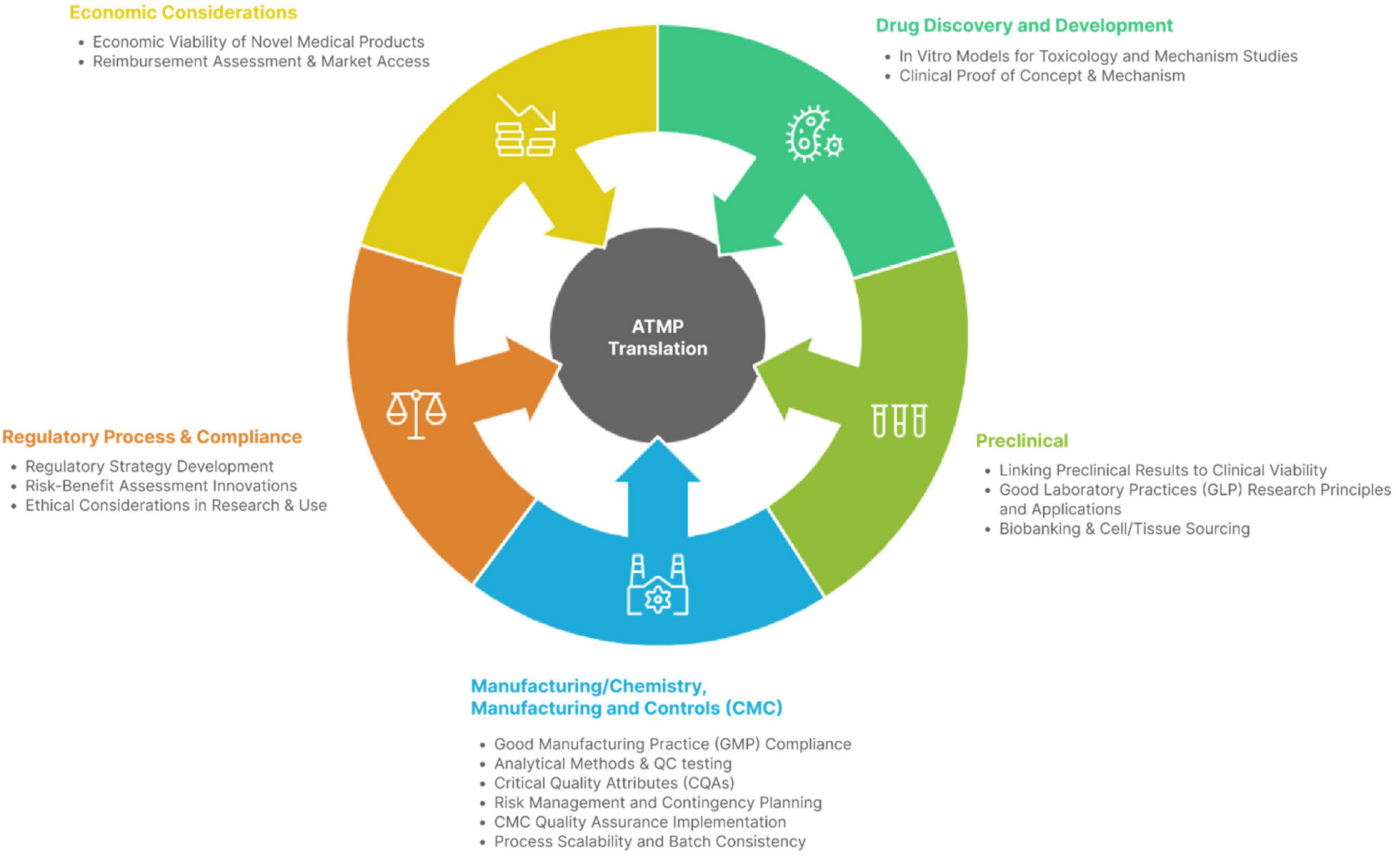
Infogram identifying competencies considered fundamental by ≥75% of experts.

Figure 1 shows the competencies where ≥75% of participants considering a competency fundamental.

It is important to note that these are not the only competencies required, nor necessarily the most important for the translation of ATMPs to the clinic. Rather, they represent the competencies that experts currently identify as key and urgently needed at this point in time.

The competencies in this final set represent the consensus core skills and knowledge areas required for effective ATMP translation, integrating regulatory, scientific, manufacturing, and economic dimensions. They reflect the points in the ATMP lifecycle where decisions most influence product viability, patient access, and regulatory success.

Together, these competencies operationalize a multidisciplinary, end-to-end approach to ATMP translation, supported by peer-reviewed evidence and expert consensus.

### 4.4 Strengths and limitations

A key strength of this study lies in its multi-phase design, combining a targeted literature review with a structured expert consensus survey. This ensured that the competencies identified are both evidence-informed and grounded in the practical experience of stakeholders directly involved in ATMP development, manufacturing, regulation, and translation. The purposive sampling approach resulted in a small sample, as only experts in translation were invited. However, this strategy enabled the recruitment of a highly specialized panel with recognized expertise, while the anonymous online format minimized dominant voices and encouraged equal contributions. Representation from academia, industry, and regulatory bodies further enhanced the robustness and applicability of the findings.

Several limitations should be acknowledged. First, the survey was conducted in English, which may have excluded experts with valuable insights who were less comfortable participating in this language. Second, although efforts were made to include diverse stakeholders, participants were predominantly from regions with established ATMP infrastructures (i.e., Europe and the US), potentially introducing geographical bias and limiting applicability to settings with different regulatory or manufacturing realities. Third, while the online format facilitated global participation, it may have limited the depth of discussion compared to interactive in-person workshops. Fourth, the purposive selection of experts, while ensuring participation of individuals with recognized experience and knowledge in the field, may introduce subjectivity and could potentially lead to selection bias. Fifth, the pre-selected list of competencies could be seen as limiting the breadth of responses and potentially anchoring participants' thinking to a preconceived set of skills and therefore may introduce potential anchoring bias. The decision to take this approach was to limit the time required for completion of the survey, to present a comprehensive list of skills for the experts to judge, and to ensure that all potentially key skills were graded by the experts. However, that selection was thoroughly based on literature research to cover all main competencies. In addition, open fields were provided for the experts to suggest additional items. No additions were registered, which indicates the initial competency list presented was valid. Sixth, while multi-round Delphi approaches are frequently used in competency framework development, a single-round structured expert consensus survey was selected in this study to maintain anonymity and maximize participation across a geographically and professionally diverse expert group. Finally, the cross-sectional design captures expert opinion at a single time point; given the rapidly evolving nature of ATMP science, emerging technologies and regulatory frameworks may shift perceptions of which competencies are considered fundamental in the near future.

### 4.5 Future directions

Now that a specialized set of competencies for ATMP translation has been identified, the next step is to build a solid delivery system through targeted education and training programs. This process could begin with a review of current offerings, identification of gaps, and definition of tailored content to address them. Educational delivery should leverage existing channels and country-specific pathways, ensuring contextual relevance.

A harmonized implementation plan, adaptable across regions, would ideally include: (i) a standardized curriculum based on the identified competencies, delivered via in-person, online, or blended formats; (ii) a common evaluation process recognized internationally; (iii) short-term placements (3–6 months) within regulatory agencies or regulatory departments of commercial developers to understand decision-making processes; (iv) short-medium-term placements in academic or industrial manufacturing facilities to observe GMP principles in practice; (v) coverage of both academic and industrial perspectives; (vi) structured mentorships and career guidance, including exposure to diverse roles in regenerative medicine; and (vii) integration of patient perspectives.

This range of competencies allows tailoring to the specific needs of different regions, institutions, and therapeutic programs. While prioritization of competencies is necessary to focus resources effectively, it is also optimal to maintain a flexible spread of options to accommodate diverse contexts and stages of ATMPs development. Moreover, regular revision of these priorities should allow to maintain alignment with real-world priorities. Although priorities would vary according to the specific therapy and the development setting, it is likely that two competencies would be required in most settings: skills to manufacture products in compliance with regulatory quality standards and the planning and execution of a clear regulatory strategy from early on. Additionally, implementation should be monitored to assess uptake and effectiveness, guiding iterative improvements.

## 5 Conclusion

Expert opinions converged on a core set of competencies with the greatest impact on translating ATMPs into the clinic, particularly in Manufacturing/CMC, regulatory strategy, and preclinical–clinical bridging. Concentrating training and development efforts in these areas is expected to accelerate the delivery of ATMPs to patients in the coming years. Achieving this goal will require strong collaboration across academic institutions, industry, and healthcare systems to establish robust educational frameworks and resources, including priority topic areas. This multidisciplinary strategic approach would also help establish a sustainable foundation for their future growth and integration into healthcare.

## Data Availability

The original contributions presented in the study are included in the article/Supplementary Material, further inquiries can be directed to the corresponding author.
