# Supplementary material for: Defining core competencies for advanced therapy medicinal products translation
Source: Front Digit Health. 2026 Feb 12;8:1707741. doi: 10.3389/fdgth.2026.1707741 (PMC12935907; doi:10.3389/fdgth.2026.1707741)
Supplement: Supplementary file 1 [file Table1.docx]

Supplementary Material

# Supplementary Data

| **THEMATIC AREA** | **COMPETENCY** | **DESCRIPTION** |
| --- | --- | --- |
| **DRUG DISCOVERY AND DEVELOPMENT** | In Vitro Models for Toxicology and Mechanism Studies | Describe importance of correlating in vitro models for applicability to toxicology, target mechanism and metabolism. |
|  | Biomarkers and Surrogate Endpoints | Identify and understand the relative utility of biomarkers and surrogate endpoints for addressing questions of efficacy and toxicity, and being able to select and improve the most adequate option for your product and patient population. |
|  | Clinical Proof of Concept & Mechanism | Define the critical parameters needed to demonstrate a valid proof of concept and proof of mechanism, highlighting the evidence generation required to validate the product’s potential. |
| **PRECLINICAL** | Linking Preclinical Results to Clinical Viability | Bridge the gap between preclinical outcomes and clinical development (i.e., formulation and clinical aspects of drug development) by integrating in vitro, in vivo, and computational models. |
|  | Preclinical Models and Alternatives | Describe selection, qualification and innovation of animal models and animal model alternatives (i.e., 3D tissue models, and organoids) to promote novel clinical trial design. |
|  | Good Laboratory Practices (GLP) Research Principles and Applications | Describe the basic principles for GLP research and when such methods are needed. |
| **CLINICAL DEVELOPMENT & TRIAL INNOVATION** | Innovative Clinical Trial Designs | Implement novel and adaptive trial designs (e.g., basket trials, synthetic controls, decentralized trials) tailored for small patient populations and individualized therapies. |
|  | Long-Term Follow-Up in Trials | Develop strategies for long-term patient monitoring, post-trial obligations, and real-world evidence generation for Advanced Therapy Medicinal Products (ATMPs). |
| **TECHNOLOGY & ANALYTICAL TOOLS** | Statistical Approaches for Novel Trial Designs | Evaluate applications of statistical approaches, biomedical informatics, and modeling (e.g., missing data handling, multiple endpoints, patient enrichment, adaptive designs) to support innovative clinical trial design. |
|  | Modeling & In Silico Methods | Leverage in silico modeling for both preclinical and clinical uses (i.e., optimize dosing, predict responses, and inform regulatory decisions and optimize trial design, including minimizing required sample size), Artificial Intelligence, machine learning, and digital twins to predict ATMP efficacy, toxicity, and immunogenicity. |
|  | Biobanking & Cell/Tissue Sourcing | Manage ethical and regulatory challenges in biobanking and sourcing of human-derived materials for ATMPs. |
|  | Emerging Technologies in Regulatory Science | Describe emerging key technology areas and their impact on regulatory science processes and policies (e.g., manufacturing, toxicology, etc.). |
|  | Platform Technology Integration | Assess the use of platform technologies to enhance scalability, cost-efficiency, and adaptability for future products. |
| **MANUFACTURING/CHEMISTRY, MANUFACTURING AND CONTROLS (CMC)** | Good Manufacturing Practice (GMP) Compliance | Ensure compliance with advanced therapies-specific GMP requirements, including autologous vs. allogeneic product considerations. |
|  | Analytical Methods & Quality Control testing | Develop robust analytical strategies for identity, potency, and stability testing in ATMPs. |
|  | Critical Quality Attributes | Identify, define, and validate Critical Quality Attributes for ensuring product safety, efficacy, and consistency. |
|  | Risk Management and Contingency Planning | Develop strategies to manage manufacturing risks and disruptions to ensure process continuity and robustness. |
|  | CMC Quality Assurance Implementation | Establish and oversee quality assurance systems with control procedures for data intake, management, and monitoring in ATMP manufacturing and CMC processes. |
|  | Process Optimization & Tech Transfer | Optimize manufacturing processes for cost, yield, efficiency, and robustness, as well as ensuring smooth technology transfer across stages and facilities. |
|  | Automation Evaluation & Implementation | Assess automation levels to minimize variability, ensuring that processes meet regulatory requirements and finalize approved protocols for seamless implementation. |
|  | Process Scalability and Batch Consistency | Assess the scalability (scale-up/scale-out) of the manufacturing process and ensure consistency and comparability across production batches. |
| **REGULATORY PROCESS & COMPLIANCE** | Regulatory Strategy Development | Develop and implement regulatory strategies tailored to ATMPs from bench to bedside, considering accelerated pathways, adaptive licensing, and global regulatory harmonization, and analyzing opportunities and challenges within the current regulatory framework. |
|  | Regulatory Science & Systems Overview | Identify approaches and techniques to address regulatory challenges, outline a research vision, and understand the relevant regulatory system and structure. |
|  | Regulatory Product Classification | Define the regulatory classification of the product and address specific considerations, such as Genetically Modified Organisms status or orphan designation. |
|  | Law & Policy Evaluation | Analyze and assess laws, regulations, and guidance specific to ATMPs. |
|  | Post-Market Compliance & Risk Mitigation | Implement pharmacovigilance, long-term follow-up, and risk management strategies specific to ATMPs. |
|  | Risk-Benefit Assessment Innovations | Develop an understanding of current risk‐benefit assessment initiatives and requirements; while identifying opportunities and challenges of implementing new approaches to risk‐benefit assessment, including for emerging innovative technologies |
|  | Ethical Considerations in Research & Use | Address ethical challenges in advanced therapy trials, such as patient consent, long-term safety, and equitable access. |
| **ECONOMIC CONSIDERATIONS** | Intellectual Property Strategy | Ensure smooth development and commercialization by identifying Intellectual Property barriers, securing patent protection for novel methodologies, and mitigating risks of unprotected Intellectual Property related to engineered cells, gene edits, and delivery vectors. |
|  | Economic Viability of Novel Medical Products | Outline aspects impacting economic viability of novel medical products, including the role for payors in coverage and reimbursement decisions. |
|  | Funding Strategy & Sustainability | Assess funding availability, type, and duration; develop plans to secure resources, ensure financial sustainability, and mitigate resource depletion. |
|  | Global Landscape & Commercialization Pathways | Assess international ATMP markets, addressing regulatory and economic challenges for commercialization (i.e., efficacy, safety, accessibility, and feasibility of current treatments worldwide, including practical aspects of administration). |
|  | Reimbursement Assessment & Market Access | Develop evidence to support payer engagement, health technology assessment (HTA), and value-based pricing (disease prevalence, economic impact on healthcare systems, and unmet medical needs). |

**Supplementary Table 1. Definitions of all competencies included in the expert consensus survey.**
